# Prevalence, indications and fetal outcomes of operative vaginal delivery in Sub-Saharan Africa, systematic review, and meta-analysis

**DOI:** 10.1186/s12905-023-02224-3

**Published:** 2023-03-09

**Authors:** Andualem Mebratu, Abbas Ahmed, Addisu Getnet Zemeskel, Asrat Alemu, Tesfaye Temesgen, Wondwosen Molla, Zerihun Figa

**Affiliations:** Dilla University College of the Health and Medical Science Department of Midwifery, PO. BOX 419, Dilla, Ethiopia

**Keywords:** Operative vaginal delivery, Instrumental delivery, Forceps, Vacuum, Indications, Fetal outcomes, Sub-Saharan Africa

## Abstract

**Purpose:**

This systematic review and meta-analysis is intended to assess the prevalence, indications, and fetal outcome of operative vaginal delivery in sub-Saharan Africa.

**Method:**

In this study, 17 studies with a total population of 190,900 were included in both systematic review and meta-analysis. Search for relevant articles was done by using international online databases (like Google Scholar, PubMed, HINARI, EMBASE, Web of Science, and African journals) and online repositories of Universities in Africa. The JOANNA Briggs Institute standard data extraction format was used to extract and appraise high-quality articles before being included in this study. The Cochran Q and I^2^ statistical tests were used to test the heterogeneity of the studies. The publication bias was tested by a Funnel plot and Egger’s test. The overall pooled prevalence, indications, and fetal outcome of operative vaginal delivery along a 95% CI using forest plots and tables.

**Result:**

The overall pooled prevalence of operative vaginal delivery in sub-Saharan Africa was 7.98% (95% CI; 5.03–10.65; I2 = 99.9%, *P* < 0.001). The indications of operative vaginal delivery in sub-Saharan African countries include the prolonged second stage of labor 32.81%, non-reassuring fetal heart rate 37.35%, maternal exhaustion 24.81%, big baby 22.37%, maternal cardiac problems 8.75%, and preeclampsia/eclampsia 2.4%. Regarding the fetal outcome, favourable fetal outcomes were 55% (95% CI: 26.04, 84.44), *p* =  < 0.56, I2: 99.9%). From those births with unfavourable outcomes, the need for the resuscitation of new-born was highest 28.79% followed by poor 5th minute Apgar score, NICU admission, and fresh stillbirth, 19.92, 18.8, and 3.59% respectively.

**Conclusion:**

The overall prevalence of operative vaginal delivery (OVD) in sub-Saharan Africa was slightly higher compared to other countries. To reduce the increased applications and adverse fetal outcomes of OVD, capacity building for obstetrics care providers and drafting guidelines are required.

## Introduction

The practice of obstetrics care was more sophisticated and improved in the last decades because of emerging technologies and high-quality trained obstetricians and other care providers. Operative vaginal deliveries (OVD) are deliveries accomplished with the use of a vacuum device or forceps devices through the application to the fetal head and outward traction generating a force that augments the maternal pushing effort to deliver the fetus [1, 2]. It is an intervention undertaken to enable better maternal and neonatal outcomes. When performed correctly in an appropriate setting by experienced and trained practitioners it usually results in a lower risk of maternal hemorrhage, prolonged hospital stay, admission to neonatal intensive care, requires reduced analgesia, expedited more quickly, and increased mother’s chance of spontaneous vaginal birth in their subsequent pregnancy [3, 4].

OVD is recommended for maternal indications like cardiac disease, severe respiratory disease, cerebral arteriovenous malformation or proliferative retinopathy, neurologic diseases such as myasthenia gravis or spinal cord injury at risk of autonomic dysreflexia, delayed progress in the second stage of labor due to malposition or inadequate fetal descent despite the maximal maternal effort and effective uterine contractions. Fetal factors for indication of the operative vaginal delivery are fetal heart rate (FHR) abnormalities, and delayed progress [5].

Nevertheless, OVD has both maternal and fetal complications. Maternal complications are more common for forceps deliveries than vacuum deliveries, when compared with a forceps delivery, a vacuum delivery appears to reduce the number of episiotomies, first- and second-degree perineal lesions, and damage to the anal sphincter [6, 7]. The risk of soft tissue trauma, newborn problems like cephalohematoma, caput succedaneum, subgeal haemorrhage, cranial injuries, jaundice, birth asphyxia, intensive phototherapy, admission to the neonatal intensive care unit, and transient brachial plexus injury was higher during use of the forceps delivery [8–11]. In addition, the problem with operative vaginal deliveries is the failure during application. Around 8.7% of vacuum fail during the application, repeated failure and application increase the risk of neonatal complications like prolonged stay in a neonatal unit, poor Apgar scores, need for intubation, and seizures [12, 13].

Avoiding routine episiotomy and practicing the use of the selective episiotomy with clinically accurate indications for women with the risk of complication, play a great role during the second stage of labor in lowering the risk of severe genital laceration and obstetric anal sphincter injuries (OASIS), postpartum haemorrhage, and postpartum period sepsis [14–16]. In addition to that, the use of the routine partograph and low-concentration epidural infusions during labor reduces the rate of forceps delivery [6].

In Latin America and the Caribbean, the magnitude of OVD range from 11% in Ecuador to 27% in Guyana. The practice of operative vaginal delivery is higher, 31% and 98% in Nepal and Cambodia, respectively [17]. Midwifery and other obstetrics care providers understaffing that couldn’t fulfill the expected standard of one-to-one in the care-providing area affect the obstetrics outcome through work overload that affects their fulfilling responsibilities [18, 19]. In sub-Saharan Africa, there is no recent and adequate data to show the overall magnitude of OVD, its indications, and fetal outcomes, despite the procedure being widely done. This can affect the understanding of the condition and the quality of obstetrics care provided in the continent. Thus, the main aim of this systematic review and meta-analysis was to assess the prevalence, indication, and fetal outcome of operative vaginal delivery in Africa.

Research questions

- What is the prevalence of operative vaginal deliveries in Africa?
- What are the indications for the obstetric intervention of operative vaginal deliveries in Africa?
- What are the fetal outcomes from operative vaginal deliveries in Africa?

## Methods

### Study setting

This systematic review and meta-analysis included only studies conducted in Africa.

### Search strategy

The search for relevant articles on the prevalence of OVD, indications, and fetal outcomes was carried out using online international databases (like Google Scholar, PubMed, HINARI, EMBASE, Web of Science, and African journals) and literature from electronics repositories of different Universities in Africa. The search was adopted according to the PICO formatting question from the database mentioned above. Including; ‘‘women’’, ‘‘delivery’’, ‘‘forceps’’, ‘‘vacuum’’, ‘‘instrumental delivery’’, ‘‘operative vaginal delivery’’, ‘‘prolonged second stage of labor’’, ‘‘fetal distress during the second stage of labor’’, ‘‘feta asphyxia’’ ‘‘big baby’’, ‘‘poor maternal pushing effort’’, ‘‘hypertensive disorder during pregnancy’’, ‘‘preeclampsia’’, ‘‘eclampsia’’, ‘‘cardiac disease’’, ‘‘failed induction’’, retroviral disease’’, ‘‘Africa’’. The MeSH engine term used for search include: ‘‘Women’’ OR ‘‘Forceps’’, OR ‘‘Vacuum’’ OR ‘‘Instrumental delivery’’, OR ‘‘Operative vaginal delivery’’, OR ‘‘Prolonged second stage of labor’’, OR ‘‘Fetal distress during the second stage of labor’’, OR ‘‘Big baby’’, OR ‘‘Poor maternal pushing effort’’, OR ‘‘Hypertensive disorder during pregnancy’’, OR ‘‘Preeclampsia’’, OR ‘‘Eclampsia’’, OR ‘‘Cardiac disease’’, OR ‘‘Failed induction’’, OR ‘‘Retroviral disease’’, OR ‘‘Sickle cell disease’’, AND Africa and other related terms.

### Eligibility criteria

#### Inclusion and exclusion criteria

Articles reported the prevalence, indications, and fetal outcome of operative vaginal delivery (OVD) in sub-Saharan African countries combined. All involved articles were checked for quality and appropriateness. So that all are of low-risk quality. Included literature and articles were only in the English language. Articles without complete abstracts or texts reported out of the scope of the outcome of interest were excluded.

#### Quality assessment

Joan Briggs Institute (JBI) cross-sectional quality appraisal checklist was used to assess the quality of the relevant studies [20]. The evaluation of each article and literature was carried out independently by five  authors (ZF, AA, AM, WM, and AG). The disagreements that happened during the evaluation process were resolved by the sixth and seventh authors (TT and AA). The consensus declares the quality and inclusion of the articles through critical. According to the JBI checklist, a cross-sectional study consists of eight items. The first item is to determine the presence of clear inclusion criteria in the article-the second is appropriateness in the description of the study subject and setting. The third item is whether the measurement of exposure is valid and reliable. The fourth is the proper description of the objective and standard criteria used. Fifth is whether the confounders were identified or not. Sixth is an appropriate strategy to handle confounders. The seventh is the reliability and validity of outcome measurement. Finally, the eighth is the relevance of the statistical analysis used. The JBI checklist value of 50% and above of the quality assessment indicators was low risk and suitable to be included in the analysis.

#### Data extraction

All the datasets were exported to Endnote version X8 software and then transferred to the Microsoft Excel spreadsheet to remove duplicated data. Five authors (ZF, AA, AM, WM, and AG) independently extracted all the relevant data using a standardized JBI data extraction format. The disagreements between reviewers were resolved by the sixth and seventh reviewers (TT and AA).

#### Measurement of outcome

This systematic review and meta-analysis study have three measurements of outcome variables. The first measurement of the outcome variable was the prevalence of OVD, while its indications and fetal outcome are the second and third measurements of outcome variables respectively.

##### Operative vaginal delivery

Was defined as assisting the delivery of the baby during the second stage of labor or after the cervix is fully dilated with aid of either a vacuum aspirator or forceps.

##### Indications of OVD

Were defined as the reasons for the application of either forceps or vacuum for delivery of fetus during the second stage of labor like the prolonged second stage of labour (PSSOL), fetal distress during the second stage of labor, big baby, poor maternal pushing effort, and hypertensive disorder during pregnancy, cardiac disease, and retroviral disease.

##### The prolonged second stage of the labor

Was defined as the labor progress taking ≥ 2 h for primiparous women and ≥ 1 h for multiparous women after the cervix is fully dilated (10 cm).

##### Fetal outcomes

Were defined as the conditions of the newborn following application of the OVD, which is either favourable or unfavourable. Unfavourable fetal outcomes include poor 1^st^ and 5^th^ minute Apgar score, admission of new-born to NICU, development of cephalohematoma, prolonged hospital stay ≥ 7 days, need for resuscitation, and others.

### Data analysis

According to Peters JL, the studies included in the meta-analysis should undergo a check for publication bias, to do this a Funnel plot and Eggers regression test [21] were used. In addition to this heterogeneity of the study was computed using Cochrane Q-test and I squared statistics to determine the effect of the single study on the finding [22]. Overall pooled analysis was conducted using a weighted inverse variance random-effects model. STATA version 16 statistical software was used to compute the analysis. Forest plot format and tables were used to present the pooled point prevalence, indications, and fetal outcome with operative vaginal delivery with a 95% of confidence interval (CI).

## Result

### Literature search result

#### Characteristics of the included studies

**Fig. 1:**
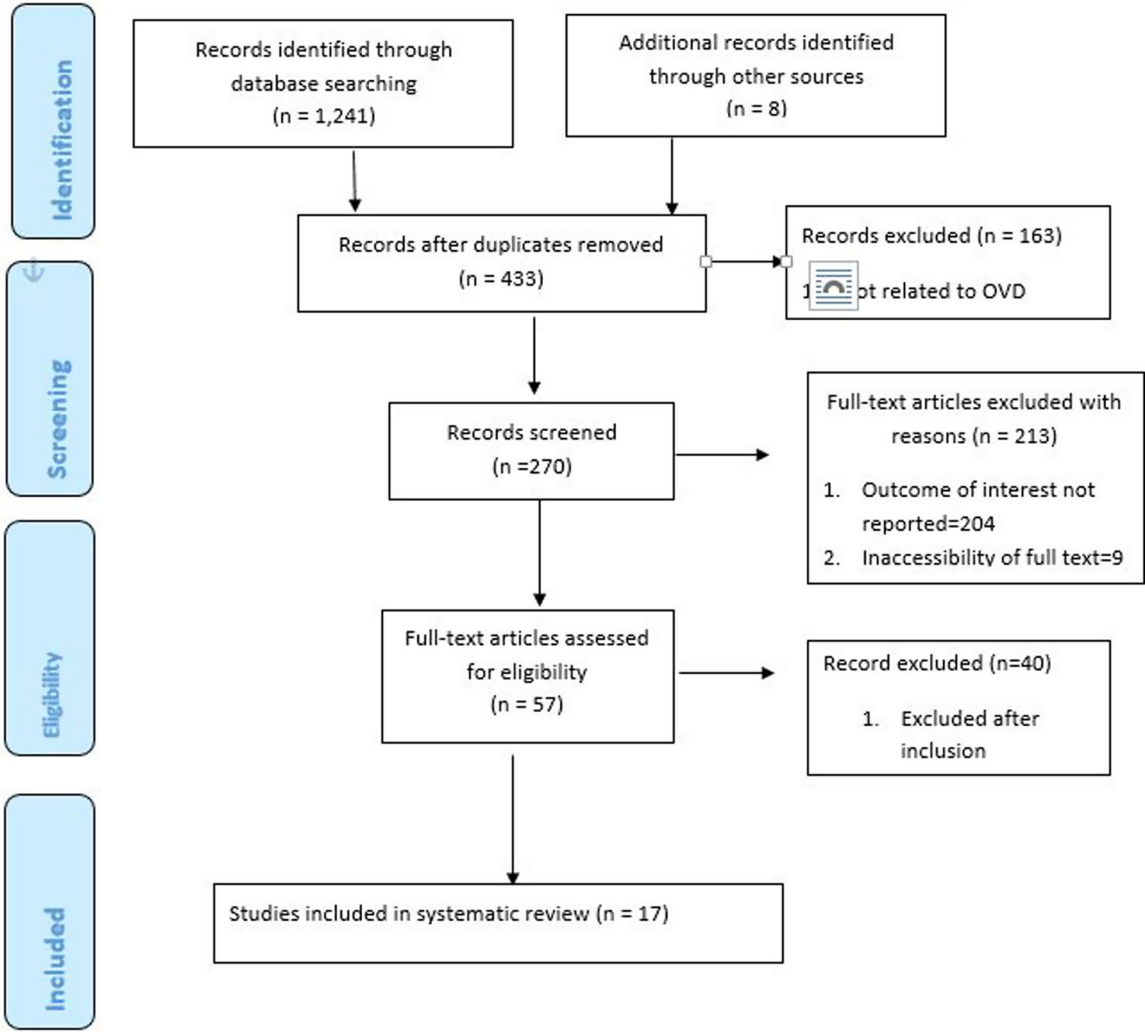
PRISMA Flow chart of study selection for systematic review and meta-analysis of prevalence, indication and fetal outcome of operative vaginal delivery in sub-Saharan African

International databases were used to search relevant articles like Google scholar, PubMed, Science Direct, web of science, HINARI, and other gray), and online repositories of Universities in Sub-Saharan Africa were used. A total of one thousand two hundred forty-nine studies published on the prevalence, indications, and fetal outcomes of operative vaginal delivery (OVD) were retrieved. After duplications were removed using Microsoft Excel, 433 studies were left for further review of their title and abstracts. Then 163 articles were excluded after a review of their titles and abstracts. Therefore, 270 full-text articles were accessed and assessed, which resulted in the further exclusion of 213 articles. From remaining 57 full-text articles were assessed for inclusion criteria. Then 40 articles were excluded because of inclusion criteria. As a result, 17 studies met the inclusion criteria to undergo the final systematic review and meta-analysis. (Fig. 1) (Table 1).

**Table 1:** Characteristics of included studies in meta-analysis of prevalence of operative vaginal delivery, indications and fetal outcome in sub-Saharan Africa

No	Author	Study year	Country	Study area	Study design	Sampling technique	Sample size	Prevalence	Prevalence of OVD		Quality
									Vacuum	Forceps	
1	Aman Yesuf et al. [5]	2016	Ethiopia	Arbamich	Cross-sectional	Consecutive	208	–	–	–	Low risk
2	Shaka et al. [23]	2019	Ethiopia	Dilla	Cross-sectional	Consecutive	2,613	8.66%	5.09%	3.51%	Low risk
3	E Nkwabong et al. [24]	2011	Cameroon	Yaoundé	Cross-sectional	Consecutive	3,623	2.30%	1.42%	0.883%	Low risk
4	Shimeles Biru et al. [25]	2019	Ethiopia	Bahridar	Cross-sectional	Consecutive	406	–	–	–	Low risk
5	Opoku [26]	2006	Ghana	Komfo Anokye	Cross-sectional	Consecutive	11,122	–	3.10%	–	Low risk
6	Hubena et al. [27]	2017	Ethiopia	Jima	Cross-sectional	Consecutive	2,348	10.3%	–	–	Low risk
7	Abegizer et al. [28]	2015	Ethiopia	Mettu	Cross-sectional	Consecutive	3,346	29.4%	21.7%	1.3%	Low risk
8	Egbodo et al.[29]	2018	Nigeria	Nasarawa State	Cross-sectional	Consecutive	7,503	0,56%	0.53%	0.03%	Low risk
9	Daru et al.[30]	2018	Nigeria	Jos	Cross-sectional	Consecutive	16,614	0.40%	–	–	Low risk
10	Kadas et al. [31]	2011	Nigeria	Bauchi	Cross-sectional	Consecutive	19,412	0.69%	0.54%	0.15%	Low risk
11	Yakasai et al. [32]	2015	Nigeria	Kano	Cross-sectional	Consecutive	22,680	–	0.9%	–	Low risk
12	Vale´rie Briand et al. [33]	2012	Senegal and Mali	–	Cross-sectional	Consecutive	78,166	12.50%	–	–	Low risk
13	Weldamanuel et al. [34]	2020	Ethiopia	Tigray	Cross-sectional	Consecutive	326	–	–	–	Low risk
14	Shiferaw and Toma [35]	2017	Ethiopia	Mizan	Cross sectional	Systematic	1854	–	11.25%	–	Low risk
15	Gebre and Hailu [36]	2017	Ethiopia	Tigiray	Cross sectional	Consecutive	357	–	–	–	Low risk
16	Adaji [37]	2009	Nigeria	Zaria	Cross sectional	Consecutive	7,327	3.6%	–	–	Low risk
17	Abebaw and Kebede [38]	2021	Ethiopia	Addis Ababa	Cross sectional	Consecutive	12,995	11.9%	4.86%	7.04%	Low risk

#### Prevalence of operative vaginal delivery in Africa

**Fig. 2:**
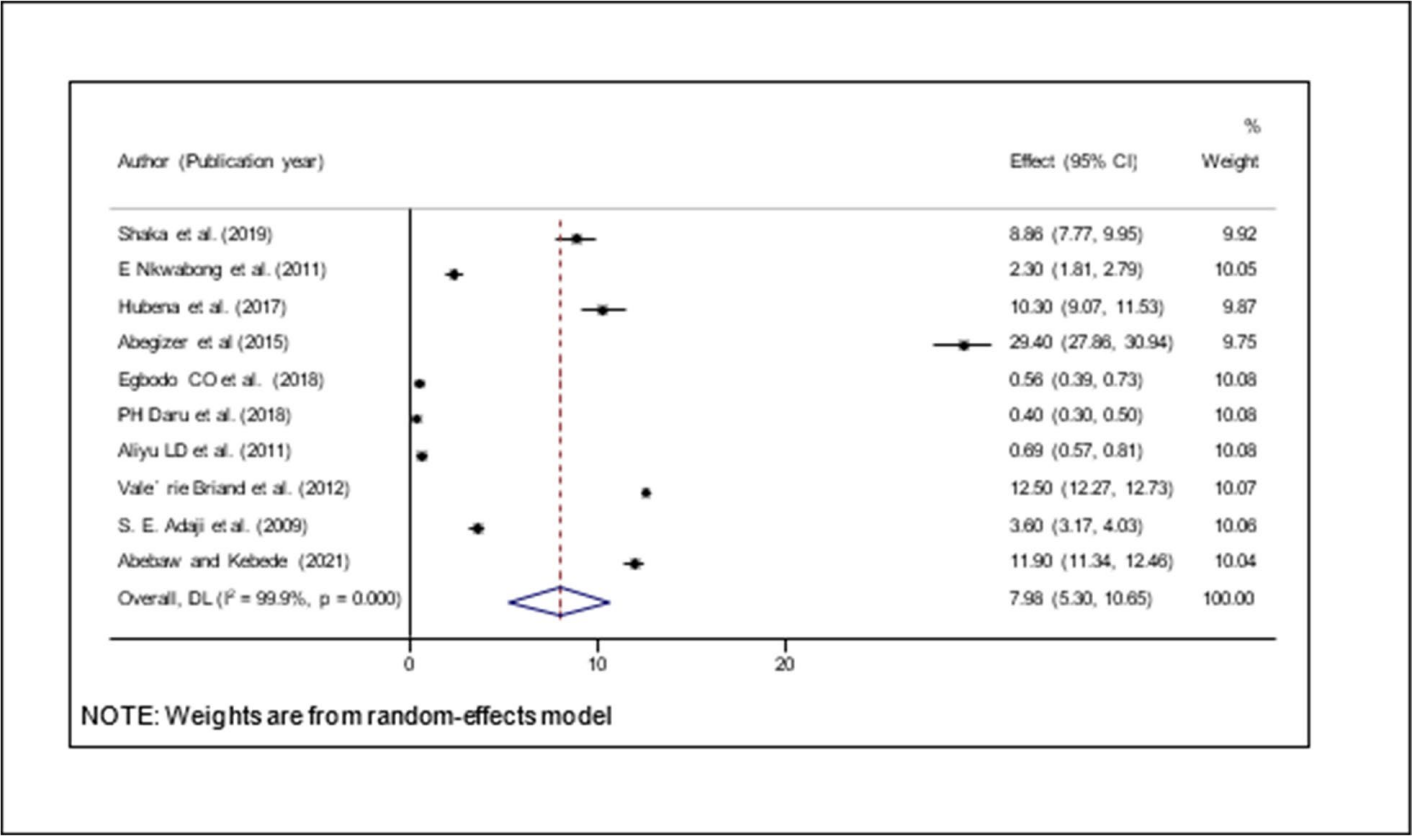
Forest plot of prevalence of operative vaginal delivery with a corresponding 95%CI of 10 studies

Ten studies were included for meta-analysis. The overall pooled prevalence of operative vaginal delivery (OVD) was presented using a forest plot. Therefore, the pooled estimated prevalence of OVD in Sub-Saharan Africa was 7.98% (95% CI; 5.03–10.65; I square = 99.9%, *P* < 0.001). (Fig. 2).

#### Publication bias

**Fig. 3:**
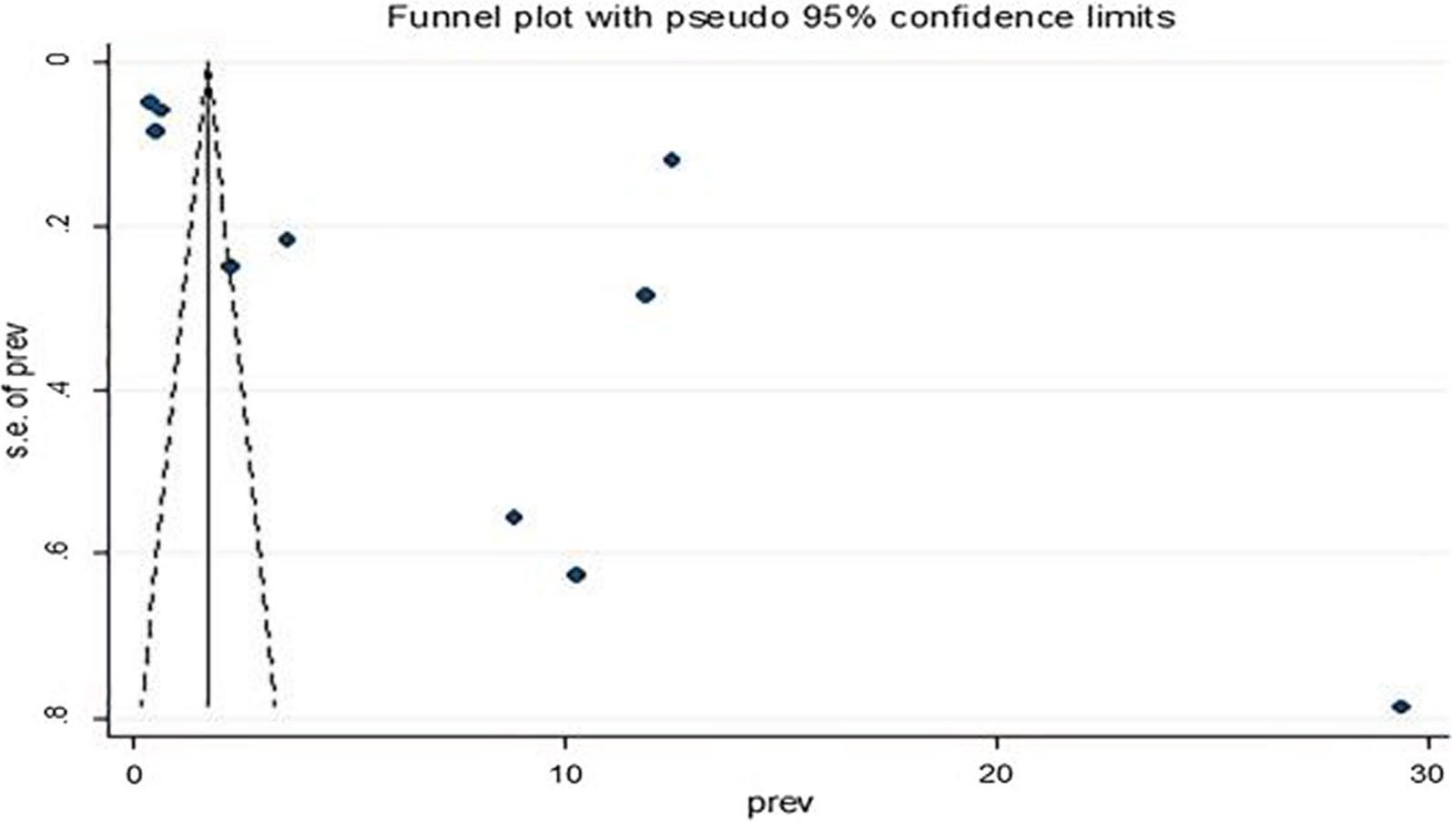
Forest plot test for publication bias for operative vaginal delivery in sub-Saharan African countries

To check publication bias a funnel plot was used. A funnel plot was inspected visually to determine the asymmetry in the distribution of the practice of operative vaginal delivery (OVD). (Fig. 3). Egger’s regression test showed a p-value of 0.066 that indicated the absence of a small study effect or publication bias.

#### Indications of operative vagina delivery practice

This study showed common indications of operative vaginal delivery (OVD) in sub-Saharan African countries, including the prolonged second stage of labor 32.81%, non-reassuring fetal heart rate pattern 37.35%, pure maternal pushing effort 24.81%, big baby 22.37%, maternal cardiac problem 8.75%, and preeclampsia/eclampsia 2.4%. (Table 2).

**Table 2:** Indications of operative vaginal delivery in sub-Saharan Africa

Indications of OVD	Model	Status of heterogeneity	Prevalence (95%CI)	I^2^ (%)	*P *value
Prolonged second stage of the labor (SSOL)	Random	Marked	32.81%(30.32, 35.24)	94.7%	< 0.01
NRFHRP	Random	Marked	37.35%(35.08, 39.63)	89.3%	< 0.01
Poor maternal pushing effort	Random	Marked	24.81%(20,32, 29.29)	43.9%	0.098
Big baby	Random	Marked	22.37%(17.71,27.01)	83.6%	< 0.01
Maternal cardiac problem	Random	Minimal	8.75%(4.55,12.95)	17.5%	0.303
Preeclampsia/eclampsia	Random	Marked	2.4%(0.48,3.60)	71.6%	< 0.01

#### Fetal outcomes following operative vaginal delivery

The overall prevalence of favourable fetal outcomes after the application of operative vaginal delivery (OVD) in Sub-Saharan African countries was 55% (95CI: 26.04, 84.44), *p* =  < 0.56, I square: 99.9%). From those births with unfavourable outcomes need for the resuscitation of new-born was highest at 28.79%, followed by poor 5th minute Apgar score, NICU admission, and fresh stillbirth, 19.92, 18.8, and 3.59%, respectively. (Table 3).

**Table 3:** Fetal outcomes following operative vaginal delivery in sub-Saharan Africa

Fetal outcome	Model	Status of heterogeneity	Prevalence (95%CI)	I^2^ (%)	*P *value
Poor 5^th^ minuet Apgar score < 7	Random	Marked	19.92%(13.72, 26.11)	89.8%	< 0.01
NICU admission	Random	Marked	18.8%(10.56, 27.05)	23.2%	0.254
New-born resuscitation	Random	Marked	28.78%(21.06, 36.51)	79.9%	0.026
Fresh stillbirth	Random	Marked	3.59% (0.78, 11.49)	0.00%	0.834

## Discussion

According to sustainable development goal 5 (SDG), maternal mortality and morbidity are high in developing countries like Africa, where poor maternity care services are provided, with limited qualified obstetrics care providers and service accessibility. To save the lives of more than half a million women who die because of complications from pregnancy and childbirth each year improving maternal health is vital. Almost all these deaths could be prevented if women in developing countries had access to adequate diets, safe water, sanitation facilities, basic literacy, and health services during pregnancy and childbirth [39].

Operative vaginal delivery (OVD) is an obstetrics intervention during the second stage of labor that helps reduce maternal and fetal complications and death. Despite its necessity and importance in obstetrics, it has its drawback for both mother and newborn that can put them in short and long-term complications. In sub-Saharan Africa, there are no adequate data on clinical practices of OVD, its indications, and feto-maternal outcomes. So, it is hard to understand the situation and plan further interventions to improve the quality of obstetrics care.

According to this systematic review and meta-analysis, the overall operative vaginal delivery (OVD) was 7.98% (95% CI; 5.03–10.65) in sub-Saharan African countries. There is one study supporting this finding from India 5.25% [40]. This similarity might be because of the similarity of the study design and both Sub-Saharan African countries and South-East Asian countries belong to low and middle-income countries. However, this finding was higher than studies conducted in Turk 1.4% [41], Nepal 2.4% [42], another study from Nepal 3.4% [43], India 1.3% [44], and a similar study from India 2.8% [45]. The justification for this variation might be because of a higher rate of caesarean section and enhanced qualification. In addition to that single study with a three times higher rate of operative vaginal delivery in Japan 18% [46]. This discrepancy might be because this stay was conducted to determine the effeteness of the guideline complaints.

This systematic review and meta-analysis showed that the PSSOL, maternal exhaustion/poor pushing effort, maternal cardiac problem, preeclampsia and eclampsia, fetal asphyxia, and big baby (> 4000 g) were the common indications for OVD in sub-Saharan Africa. This finding was supported by the study from Turk [41], Nepal [42], India [47], another study from India [48], Russia [49], and the United Kingdom [44].

Regarding fetal outcome, following the application of either vacuum or forceps. The common unfavourable fetal outcomes are poor 5th minute Apgar score, admission to NICU, need for the new-born resuscitation, and fresh new-born. This finding was supported by the study conducted in Russia [49], India [40], Greece [50], another study in India [45], Pakistan [51], and Nepal [43]. The presence of significant heterogeneity in this systematic review and meta-analysis may expose the finding to publication bias. This might be due to the sample size of each study, the nature of the study design, incomplete data, and the study settings. The overall magnitude of operative vaginal delivery (OVD) in Sub-Saharan African countries is 7.98% which is slightly higher compared to other developed countries. This could be because of existing policy and policy gaps to reduce the practice.

## Conclusion

The overall prevalence of operative vaginal delivery (OVD) in sub-Saharan Africa was higher compared to other countries. It is the cause of complications and morbidity in new-born. To reduce increased applications of OVD and poor fetal outcomes, capacity building for obstetrics care providers and drafting guidelines are required.

## Strength of study

This review showed current obstetrics practice, its indications, and the fetal outcome of operative vaginal delivery in sub-Saharan Africa. We hope this will help to increase understanding of current obstetrics practices.

## Limitation study

All of the included studies in this systematic review and meta-analysis were conducted using a retrospective cross-sectional study design which has a limitation on the quality of data and completeness of documentation. There may be more chances to do these types of studies in institutions with higher rates of OVD in sub-Saharan Africa. Also, it may lack representativeness because the included data was only from 5 countries of sub-Saharan African countries.

## Data Availability

All related data has been presented within the manuscript. The dataset supporting the conclusions of this article is available from the correspondent author at a reasonable request.

## Abbreviations

CI: Confidence Interval
OVD: Operative vaginal delivery
PSSOL: The prolonged second stage of labor
OR: Odds Ratio
JBI: Joan Briggs Institute: Sever complication obstetric anal sphincter injuries (OASIS) NRAFHP: non-reassuring fetal heart rate pattern
